# Optimization of antibiotic selection in the emergency department for urine culture follow ups, a retrospective pre-post intervention study: clinical pharmacist efforts

**DOI:** 10.1186/s40545-019-0168-z

**Published:** 2019-04-09

**Authors:** Abdulaziz Saleh Almulhim, Ali Aldayyen, Kateryna Yenina, Adam Chiappini, Tahir Mehmood Khan

**Affiliations:** 1 King Faisal University, College of Clinical Pharmacy, Al-Ahsa, Saudi Arabia; 2 The University of Arizona, Department of Pharmacy Practice, College of Pharmacy, Tucson, AZ USA; 3 Banner-University Medical Center South, Pharmacy Department, Tucson, USA; 4 Monash University, School of Pharmacy, Clayton, Australia; 5 Institute of Pharmaceutical Sciences, University of Veterinary and Animal Sciences, Outfall Road, Lahore, Pakistan

**Keywords:** Urinary tract infection, Pharmacist, Emergency Department, Antimicrobial stewardship

## Abstract

**Introduction:**

Urinary tract infections (UTI) are commonly encountered in the emergency department (ED). ED culture follow up is an important tool to provide the appropriate therapy after the identification of the causative pathogen. There is a growing body of evidence for the positive role of pharmacists in following up the ED cultures. The purpose of this study was to compare pharmacist driven urine culture follow ups to the nurse-practitioner (NP) driven follow ups in term of the appropriateness of antibiotic selections in patients with resistant isolates, inappropriately treated asymptomatic bacteriuria, and inappropriately chosen antibiotic.

**Methodology:**

This was a retrospective pre-post intervention study divided into a two group period to compare pharmacist to NP led ED culture follow up interventions. Statistical Package for Social Sciences (SPSS) version 20 was used for analysis. Student’s *t*-test was used for continuous variables and Chi-square test/or fisher’s-exact test when appropriate were used for the primary outcome.

**Results:**

Fifty-five patients (25.7%) and 102 (34%) met the inclusion criteria in the pharmacist arm and in the NP arm, respectively. *Escherichia coli* was the most commonly isolated pathogen in both arms. Asymptomatic bacteriuria was often treated in the ED in both groups (45/157, 28.7%) and there were no efforts in discontinuation of antibiotics in these patients. Neither the interventions group nor the no interventions groups were statistically different between the pharmacist and NP arms (*P* 0.0778), (*P* 0.797), respectively.

**Conclusion:**

No statistically significant difference was observed between pharmacist driven monitoring and NP driven monitoring. In our institution, asymptomatic bacteriuria was commonly treated even in the absence of indications. We recommend that Pharmacists’ roles in the ED cultures follow up be expanded to include antibiotic discontinuation in patients who meet asymptomatic bacteriuria criteria or have confirmed negative urine culture.

**Electronic supplementary material:**

The online version of this article (10.1186/s40545-019-0168-z) contains supplementary material, which is available to authorized users.

## Background

Urinary tract infections (UTIs) are one of the most commonly encountered infections in the emergency department (ED) [1]. It is estimated that UTI resulted in 2–3 million visits to ED [2]. The clinical presentation of UTI ranges from uncomplicated cystitis to complicated pyelonephritis and sepsis [3]. Moreover, UTI can be classified as asymptomatic bacteriuria, which is defined as the quantitative isolation of bacteria in an appropriately collected urine specimen in the absence of signs and symptoms [4]. While treatment of UTI depends mainly on the classification and severity, treatment of asymptomatic bacteriuria is unnecessary except in certain populations e.g., pregnancy and prior to surgical urological procedures [5]. Despite the availability of guidelines, asymptomatic bacteriuria is frequently treated [6, 7].

The inappropriate use of antibiotics was first recognized in the early 1940s. Issues associated with the inappropriate or unnecessary use of antibiotics include but are not limited to the following: selection of resistance, secondary infections, and adverse drug reactions [8]. To limit these problems, antimicrobial stewardship programs (ASP) were established. The focus of these programs is to limit the unnecessary exposure to antibiotics, improve clinical outcomes, reduce the resistance rate and decrease the financial burden [9]. Given the importance of ASP in the inpatient setting and given the fact that implemented changes to antimicrobial therapies initiated in the ED creates a challenge for ASP team, several institutions have implemented the ASP services in the ED [7, 10, 11]. There is a growing body of evidence for the positive role of pharmacists in the ED and reduced hospital admissions [10, 12, 13]. Davis et al. reported that the introduction of Clinical Pharmacist positive culture follow ups in ED led to an absolute increase in 30% in interventions for inappropriate therapy [12]. Randolph et al. compared the unplanned readmission between physician-managed ED cultures and pharmacist-managed ED cultures. The readmissions were significantly lower following the implementation of pharmacist-managed ED culture review process (19 and 7% *P* < 0.001, respectively) [13].

The American Society of Health-System Pharmacists (ASHP) has recommended that every hospital pharmacy department should provide pharmacy services to it’s ED. Services include but are not limited to the collaboration with health care professionals to promote the safe and effective use of medications [14]. Based on this recommendation and the growing body of evidence for the positive role of pharmacists, our pharmacy department has taken the lead of culture follow ups in March 2017. Due to the lack of UTI guidelines in our institution (Banner-University Medical Center South), and the recent implementation of pharmacist driven culture follow up, we decided to conduct this retrospective study to evaluate the performance of our ED pharmacist urine culture follow up and compare it to the nurse practitioner (NP)-driven follow up in term of the appropriateness of chosen antibiotics and, inappropriately treated asymptomatic bacteriuria. Although fluoroquinolones are commonly prescribed antibiotics for UTIs, resistance to this class ranges from 22 to 75% depending on the country and region [15, 16]. Given the lack of resistance rates of uro-pathogens in our institution, the percentages of uro-pathogens resistant to specific antibiotic classes (etc. Sulfamethoxazole-Trimethoprim, and fluoroquinolones) were measured as a secondary outcome.

## Methods

### Study design, data source, and data collection

This was a retrospective pre-post intervention study of adult patients who visited the ED between December 2016 and June 2017 who had urine cultures performed for evaluation of urinary tract infection. The period from 4th of December to March 3rd represented NP culture follow up and interventions; the period from March 7th to June 4th represented pharmacist culture follow up and interventions. Patients were identified using International Classification of Diseases, tenth revision (ICD-10) codes for UTI. The following are the ICD-10 codes used for patient’s identification (N39.0, O86.20, Z87.440, A56.00, O23.93, O23.92, O23.91, O23.90, T83.51, O23.33, O23.32, O23.31, O23.30, O23.43, O23.42, O23.41, O23.40, O08.83, O03.38, O04.88) from our electronic health medical records system (EPIC). Banner-University Medical Center South is a tertiary care teaching hospital with an annual ED visits of 45,000. The following authors (A.S.A, A.A) reviewed charts to determine the eligibility of patients to be included in the study. In addition, both authors recorded patient’s demographics, laboratory and microbiological results, and antibiotics administered while in ED and/or prescribed after discharge. After literature appraisal, a structured data collection tool (Excel) was used to perform data collection. Moreover, few additional items were also added during the internal validity exercise conducted by the research team. Relevant data that was collected is shown in the tables presented in the result section. Patients were excluded if they were less than 18 years old, had a concomitant infection/sexual transmitted disease, UTI diagnosed prior to the ED visit, admitted to the hospital or transferred to a different facility. (Additional file 1) displays the ED cultures follow up process for both pre and post intervention periods. This study was approved by Banner-University Medical Center Institutional Review Board (IRB).

### Definitions

#### UTI

Isolation of a specified quantitative count of bacteria in an appropriately collected urine specimen obtained from a person with signs or symptoms consistent with urinary tract infection.

#### Asymptomatic bacteriuria

Isolation of a specified quantitative count of bacteria in an appropriately collected urine specimen obtained from a person without symptoms or signs consistent with urinary tract infection.

#### Appropriate/inappropriate intervention

Discontinuation of antibiotic if met asymptomatic bacteriuria criteria.

Discontinuation/changing of antibiotic based on final urine culture (negative urine culture/mixed flora).

Follow up call or certified mail letter for uncovered microorganism.

### Statistical analysis

The collected data was analyzed by using Statistical Package for Social Sciences (SPSS) version 20. Both descriptive and inferential statistics were used to analyze the data. Continuous variables were described as means ± standard deviation (SD), categorical variables were described as percentages. Chi-square test/or Fischer’s exact test when appropriate were used to compare categorical variables. Student’s *t*-test was used to compare continuous variables. All *p*-values less than 0.05 were considered statistically significant.

## Results

**Fig. 1:**
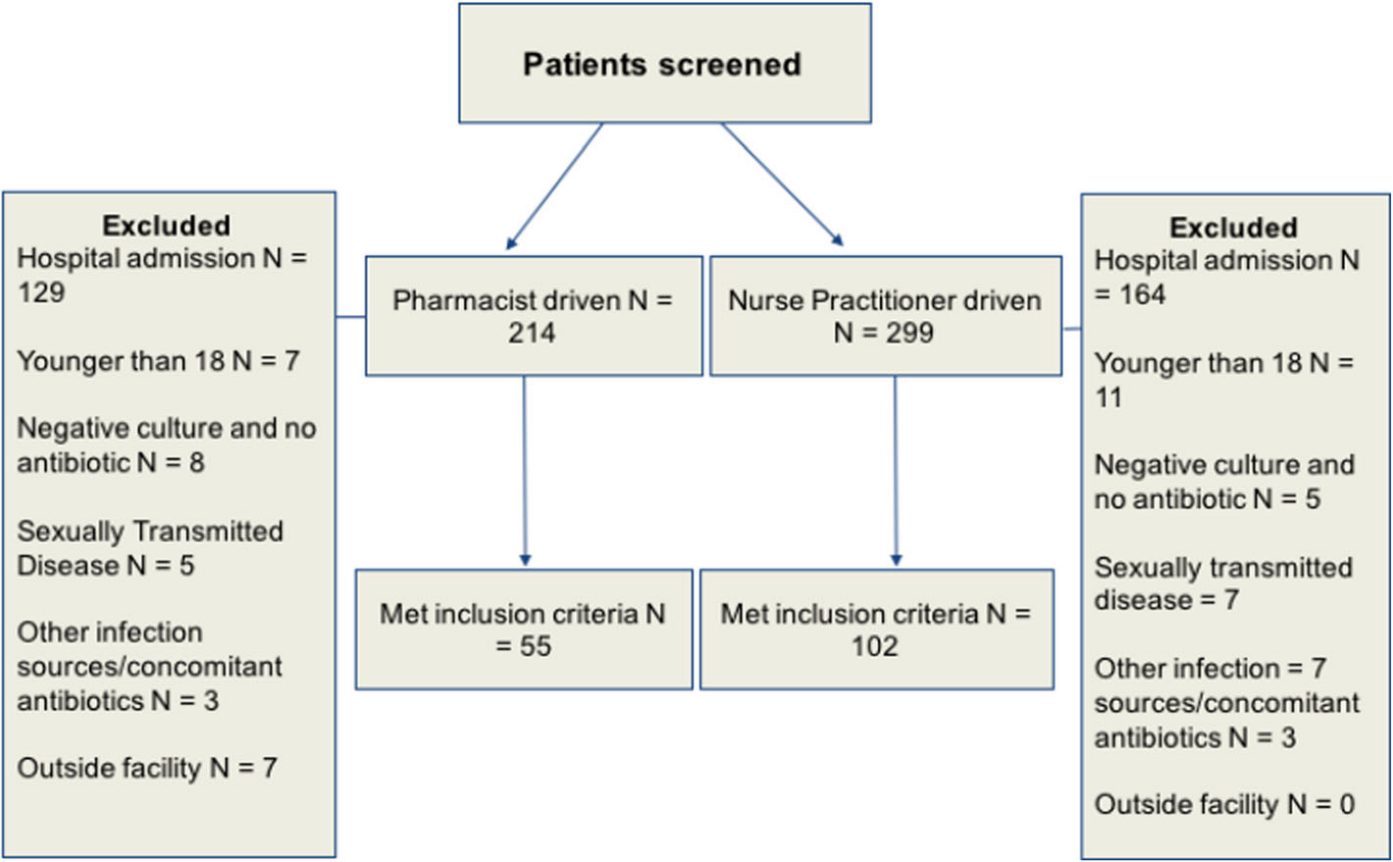
Patients screening and selection

There were 513 patients visited the ED during the pre-specified period, of which 356 were excluded from the analysis after applying the prespecified exclusion criteria. Fifty-five patients (25.7%) and 102 (34%) met the inclusion criteria in the pharmacist arm and in the NP arm, respectively (Fig. 1). The mean age was 50 and 48 years in the pharmacist arm and NP arm, respectively. Females constituted the majority of the cohort, 43 (78%) in the pharmacist arm and 82 (80%) in the NP arm. *Escherichia coli* (*E. coli*) was the most commonly isolated urinary pathogen. The majority of patients had unspecified UTI diagnosis based on complexity or type (Table 1). The most commonly prescribed antibiotic was ciprofloxacin (22/49, 45%; 32/90, 36%) in the pharmacist arm and NP arm, respectively (Table 1).

**Table 1:** Patients Demographics

	Pharmacist arm (*n* = 55)	NP arm (*n* = 102)	*P* value
Age (years), (Mean, SD)	55.05 (22)	48.04 (22)	0.0587
Sex, n (%)			
Male	12 (21)	20 (20)	0.742
Female	43 (78)	82 (80)	
Serum WBC, (Mean, SD)	8.71 (3.59)	7.59 (4.01)	0.085
Urinalysis			
Leukocyte esterase, (Mean, SD)	326.02 (205.33)	284.63 (207.37)	0.233
Urine WBC, (Mean, SD)	87.31 (168.28)	192.66 (470.37)	0.110
Urinary pathogens			
E.coli	18 (33)	24 (23.5)	
Mixed flora	19 (34.5)	22 (21.6)	
Klebsiella pneumoniae	3 (5.5)	5 (4.9)	
Pseudomonas aeruginosa	1 (1.8)	0	–
Staphylococcus aureus	1 (1.8)	6 (5.9)	
Lactobacillus	1 (1.8)	5 (4.9)	
Serratia marcescens	1 (1.8)	2 (2)	
Group B beta hemolytic streptococcus	2 (3.6)	4 (3.9)	
Type of UTI, n (%)			
Unspecified UTI	28 (50.9)	53 (52)	
Pyelonephritis	13 (23.6)	24 (23.5)	
Complicated pyelonephritis	7 (12.7)	0	–
Complicated cystitis	4 (7.3)	9 (8.8)	
Uncomplicated cystitis	2 (3.6)	16 (15.7)	
Catheter associated UTI	1 (1.8)	0	
Antibiotics, n (%)			
Ciprofloxacin	22 (45)	32 (36)	
Cefdinir	6 (10.9)	9 (8.8)	–
Cephalexin	12 (21.8)	31 (30.4)	
Nitrofurantoin	7 (12.7)	18 (17.4)	
Sulfamethoxazole-Trimethoprim	2 (3.6)	0	

*E. coli*: *Escherichia coli, UTI* urinary tract infection

**Fig. 2:**
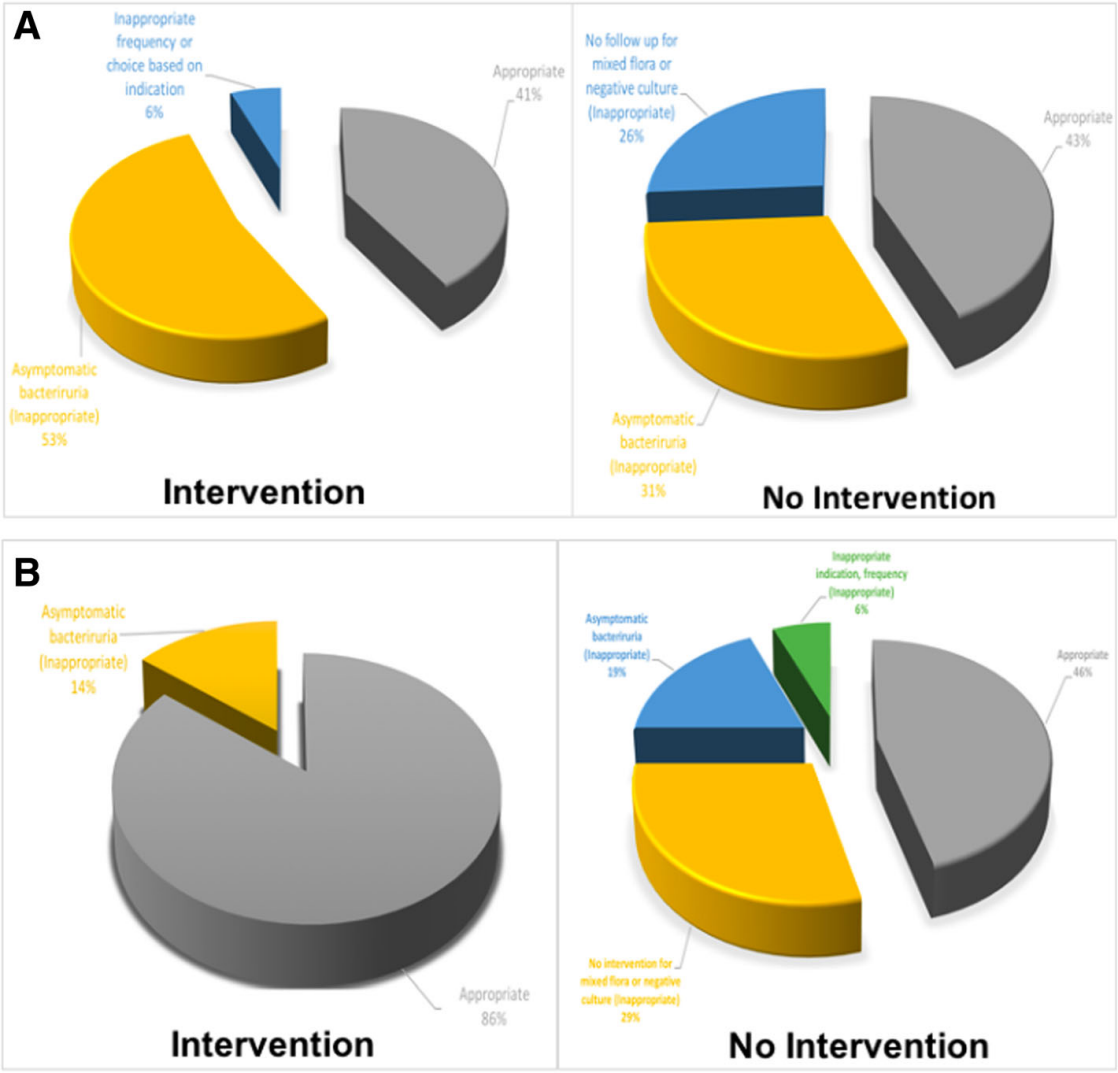
**a** Nurse practitioner arm, **b** Pharmacist arm

In the pharmacist arm, there were seven interventions, of which six were deemed appropriate. Only one inappropriate intervention was done as patient met the asymptomatic bacteriuria criteria. In the NP arm, there were 17 interventions, of which only seven were deemed appropriate. The other 10 interventions were deemed inappropriate as nine met the asymptomatic bacteriuria criteria and one patient had nitrofurantoin recommended for pyelonephritis (*P* 0.0778), (Table 2) (Fig. 2). Twenty-two patients didn’t require interventions in the pharmacist arm, compared to 37 in the NP arm as the initial intervention done by the treating physician was considered appropriate. Twenty-six patients in the pharmacist arm and 48 patients in the NP arm had inappropriate no intervention based on the pre-specified appropriateness criteria (*P* 0.797), (Table 2) (Fig. 2). The resistance rate of *E. coli* to ciprofloxacin and Sulfamethoxazole-Trimethoprim was 5.7 and 28.5%, respectively (Table 3).

**Table 2:** Primary Outcome

	Pharmacist (*N* = 55)	NP (*N* = 102)	*P* value
Intervention, n (%)	7 (13)^*^	17 (17)^*^	
Appropriate	6 (86)	7 (41)	0.0778^∋^
Inappropriate	1 (14)^a^	10 (59)^a,e^	
No Intervention, n (%)	48 (87)^*^	85 (83)^*^	
Appropriate	22 (46)^b^	37(44)^b^	0.797^∋^
Inappropriate	26 (54)^c,d,e^	48 (56)^c,d^	

^*^Percentage based on total patients per arm

^∋^Chi-square was used to assess association between variables, if cell count was less than 5 in 25% of the cells Fischer’s exact test was used

^a^Asymptomatic bacteriuria

^b^No change in therapy needed

^c^No follow up call or letter upon final negative or mixed flora urine culture

^d^Continuation of antibiotic despite asymptomatic bacteriuria

^e^Inappropriate frequency or choice based on indication

**Table 3:** Secondary outcome

E.Coli, *N* = 35	Ciprofloxacin, n (%)	Sulfamethoxazole-Trimethoprim, n (%)
Sensitive	33 (94%)	25 (71%)
Resistant	2 (5.7%)	10 (28.5)

## Discussion

In this study, the treatment of asymptomatic bacteriuria was surprisingly common (45/157, 28%) in both arms. There was no statistical difference between the pharmacist and NP interventions (*P* 0.0778). There was a trend toward more inappropriate interventions to treat asymptomatic bacteriuria in the NP arm. However, this did not reach statistical significance; likely due to small sample size. The majority of these asymptomatic bacteriuria cases were in the inappropriate no intervention group (34/133, 25.5%) (Table 2). Although current guidelines discourage the treatment of asymptomatic bacteriuria, antibiotics are still being prescribed for this population [7]. Moreover, treatment of asymptomatic bacteriuria can lead to complications. A prospective study found that treatment of asymptomatic bacteriuria in women who had prior UTI within 12 months, increased their risk of recurrent UTI when compared to women who didn’t get treatment for asymptomatic bacteriuria [8]. Although, we didn’t examine factors that triggered emergency department physicians to treat asymptomatic bacteriuria, we believe that abnormal urinalysis was the primary driving factor. Zhang et al. [7] examined factors associated with prescribing antibiotics in asymptomatic bacteriuria patients. They found that the presence of positive leukocyte esterase and nitrites in the urine were the triggers for prescribing antibiotics in asymptomatic patients. Although not clearly documented, positive urinalysis may have also played a role in NP decision to treat asymptomatic patients in this study.

In addition to abnormal urinalysis, urinary cloudiness or darkness shouldn’t be the only reasons to treat asymptomatic bacteriuria. The Canadian Association for Clinical Microbiology and Infectious Diseases recently published a position statement discouraging treating cloudy or foul smelling urine in the absence of symptoms [17]. Our current institution’s Standard Operating Procedure (SOP) doesn’t require the pharmacist to recommend antibiotic discontinuation in patients with asymptomatic bacteriuria (Additional file 1). This likely resulted in no interventions conducted in patients who were discharged on antibiotics and met the asymptomatic bacteriuria criteria.

Additionally, our SOP indicates that no action is needed if final culture positive for mixed flora. Again, this likely was the main reason no actions were performed upon final culture evaluation. The current staffing model for ED pharmacists may not provide the time required to intervene on mixed flora, negative culture results and/or asymptomatic bacteriuria.

The most commonly prescribed antibiotic class was fluoroquinolones in both arms (Table 1). The overall prescription pattern showed 54 cases treated with ciprofloxacin (34.4%). This is consistent with previous studies [18, 19]. Kobayashi et al. analyzed the National Ambulatory Medical Care and National Hospital Ambulatory Medical Care Survey datasets from 2006 to 2011. They found that fluoroquinolones were the most commonly prescribed antibiotics (49%) [18]. Our secondary outcome was to determine the incidence of *E. coli* species resistant to this class to determine if this class should still be prescribed [4]. The resistance rate was less than 10% (2/35, 5.7% in our population) (Table 3), indicating that this class can still be considered as empiric treatment in patients with pyelonephritis or complicated cystitis. However, the United States Food and Drug Administration (FDA) released a safety announcement and warned of potential disabling adverse effects with fluoroquinolones use [20–22]. In addition, the FDA advised to restrict fluoroquinolones in certain infectious disease states; uncomplicated cystitis is one of these restricted indications. In our study, we identified 18 patients diagnosed with uncomplicated cystitis. Of those, 6 patients (33.3%) were treated with fluoroquinolones (five cases discharged from ED with ciprofloxacin prescription and one case the antibiotic therapy was changed to ciprofloxacin after nurse practitioner culture review and follow up). The pharmacy department in our institution conducted a medication use evaluation in response to the FDA fluoroquinolones warning and disseminated a memo before the period of this study that include a discouragement of fluoroquinolones use in UTI especially the uncomplicated cystitis. Based on our unpublished results, more education is needed to reinforce the restriction on fluoroquinolones prescription.

This study has several limitations. First, due to the retrospective nature of our study, the accuracy of data was dependent on the quality of the documentation. For example, to define asymptomatic bacteriuria we relied on the history of present illness, review of systems and finally assessment, plan, and recommendations from the treating physician. Therefore, If UTI signs and symptoms were present but not documented, this can result in undertreating such patients. Nevertheless, this can be avoided through patient communication and verifying the presence or absence of UTI symptoms before final recommendations are made. Second, the majority of our cohort had a UTI of unspecified type (cystitis vs pyelonephritis) which limited us from evaluating the appropriateness of recommended antibiotic and duration for the specific indication. Finally, due to the recent implementation of pharmacist services in the ED we were not able to look at a longer interval, which resulted in a smaller sample size and eventually potential for Type II error in the intervention arm. However, this may not be the case as mentioned earlier that intervention is not required per our SOP guidelines.

## Conclusion

Our study showed that treating asymptomatic bacteriuria is still an ongoing problem with little if any significant interventions. Our current SOP doesn’t provide a clear recommendation to the pharmacists of what actions to be considered when asymptomatic bacteriuria is encountered. The authors recommend that an additional action to be considered to minimize and potentially completely avoid treatment of asymptomatic bacteriuria. Additionally, given the recent FDA warning, we recommend against the common and unjustified use of fluoroquinolones when alternatives are available in patients with uncomplicated cystitis. Given that this was a single-center study, the generalization of our results may not be applicable. Close attention to the resistance rate in other institutions is advised.

## Additional file

Additional file 1: Cultures workflow algorithm. (DOCX 808 kb)
