# Supplementary figures and images for: Optimization of antibiotic selection in the emergency department for urine culture follow ups, a retrospective pre-post intervention study: clinical pharmacist efforts
Source: J Pharm Policy Pract. 2019 Apr 9;12:8. doi: 10.1186/s40545-019-0168-z (PMC6454615; doi:10.1186/s40545-019-0168-z)

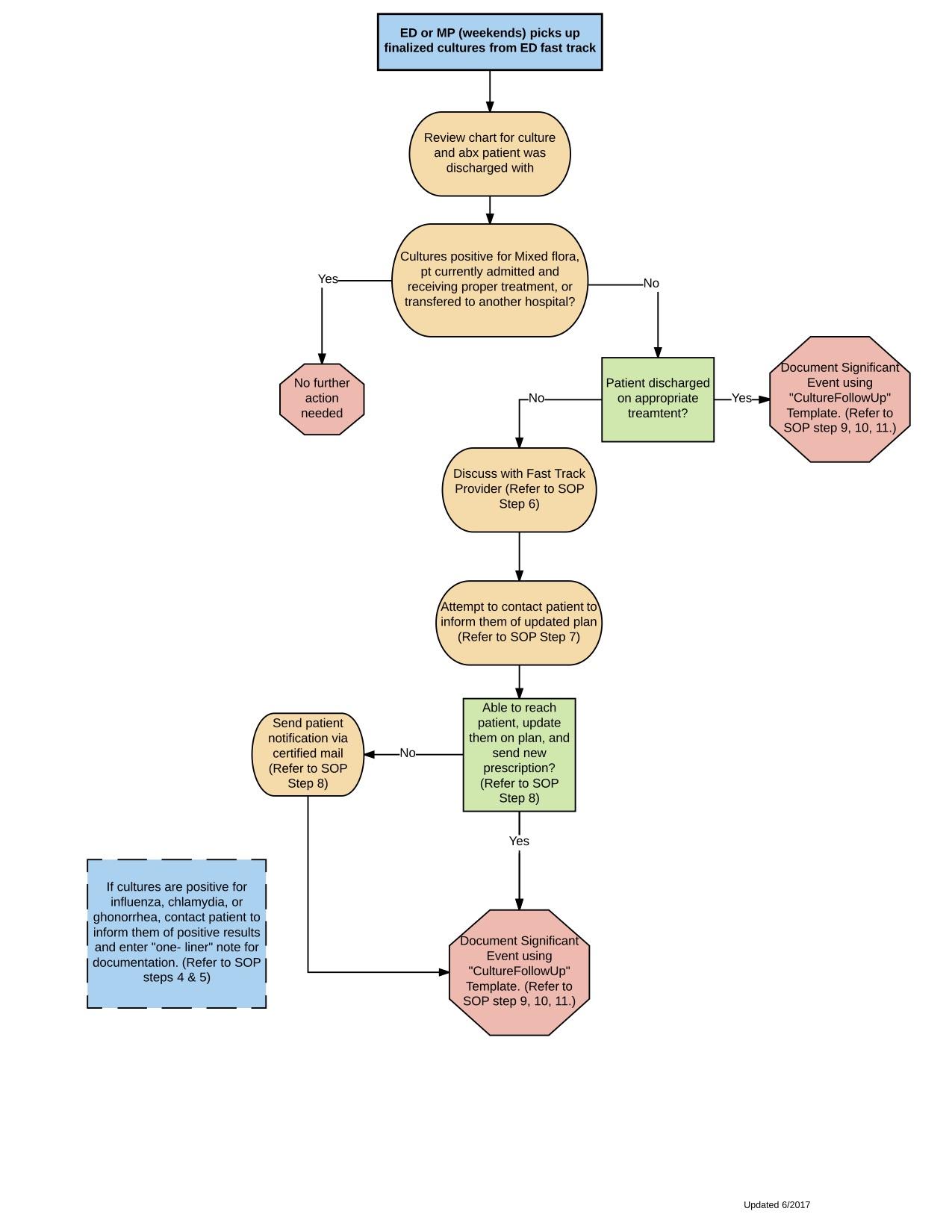


Additional file 1

Supplement: Supplementary file 1 — Cultures workflow algorithm. (DOCX 808 kb) [file 40545_2019_168_MOESM1_ESM.docx]
